# Correlation between Insulin Resistance and Thyroid Nodule in Type 2 Diabetes Mellitus

**DOI:** 10.1155/2017/1617458

**Published:** 2017-10-12

**Authors:** Yunzhao Tang, Tiantian Yan, Gang Wang, Yijun Chen, Yanjuan Zhu, Zhenhuan Jiang, Min Yang, Chenguang Li, Zhu Li, Ping Yu, Shanshan Wang, Nannan Zhu, Qiuyue Ren, Changlin Ni

**Affiliations:** ^1^Key Laboratory of Hormones and Development (Ministry of Health), Tianjin Key Laboratory of Metabolic Diseases, Tianjin Metabolic Diseases Hospital & Tianjin Institute of Endocrinology, Tianjin Medical University, Tianjin, China; ^2^Tianjin University of Traditional Chinese Medicine, Tianjin, China

## Abstract

**Objective:**

The present study explored the association between insulin resistance (IR) and the clinical characteristics of thyroid nodules in patients with type 2 diabetes mellitus (T2DM).

**Methods:**

All the patients were newly diagnosed with T2DM. 201 patients with thyroid nodule disease and 308 patients without the nodular thyroid disease. The participants were evaluated by relevant examination. Correlation analyses and regression analyses were performed to examine the relationships between the two groups.

**Results:**

HOMA-IR values, serum FT4 (free thyroxine) levels, and age were higher in the thyroid nodule group than in the control group. The proportion of women in the thyroid nodule group is greater than the proportion of women in the control group. Logistic regression analysis showed that age, sex, FT4, and HOMA-IR were positive factors for thyroid nodule. The volume and size of the thyroid nodule were positively correlated with HOMA-IR, irrespective of gender. The thyroid nodule volume and size and the TSH (thyroid stimulating hormone) were greater in females than in males, whereas FT3 (free triiodothyronine) was lower in females.

**Conclusion:**

IR might be a risk factor for thyroid nodule. Whether alleviating the IR might slow the growth, or diminish the volume and size of the thyroid nodules, is yet to be elucidated.

## 1. Introduction

IR is a risk factor for several diseases such as coronary artery disease, polycystic ovarian syndrome, essential hypertension, nonalcoholic fatty liver disease, and other disorders [1, 2]. One of the characteristics of a large number of patients with type 2 diabetes mellitus (T2DM) is the presence of IR within target tissues and the response of hyperinsulinemia [3]. Insulin is known to act as a growth factor that stimulates cell proliferation. Previous studies reported that, as a comparison to the nonthyroid nodule patients, the thyroid nodule patients exhibited a higher homeostasis model assessment-IR (HOMA-IR) value in patients with normal glucose metabolism [4]. On the other hand, greater thyroid volume and higher nodule prevalence were seen in patients with impaired glucose metabolism [5–7]. However, few studies have focused on the relationship between thyroid nodules and IR in patients with T2DM. A number of well-known factors are involved in nodule formation including iodine deficiency, gender, age, smoking, and genetic factors [8, 9]. International Diabetes Federation (IDF) announced a continual increase in the rate of diabetes around the world. Similarly, the incidence of thyroid nodule was also rising. Thus, the characteristic features of T2DM, such as IR, might be associated with an increased risk. In this study, we aimed to investigate whether the IR plays a role in the thyroid nodule in patients with T2DM.

## 2. Material and Methods

### 2.1. Subjects and Study Design

A prospective cross-sectional study was performed from March 2014 to 2016. All patients were recruited from the Tianjin Metabolic Diseases Hospital and diagnosed with T2DM according to the guidelines for type 2 diabetes in China, 2013 [10], had not received any oral antidiabetic drugs or insulin treatments, and showed normal thyroid function. And the body mass index (BMI) was recorded between 22 and 45 kg/m2 at the first visit. Exclusion criteria are abnormal thyroid function, iodine deficiency (urinary iodine concentration < 100 *μ*g/L), iodinated contrast material exposure in the previous 6 months, history of neck radiation treatment or surgery, diagnosis of type 1 diabetes mellitus, history of heart failure with an ejection fraction < 30% or NYHA classification > 2, history of liver diseases such as cirrhosis, hepatitis B, or hepatitis C (except carriers), alanine aminotransferase (ALT), aspartate aminotransferase (AST) greater than two times the upper limit of normal (ULN) or total bilirubin greater than 2-fold ULN, and clinical diagnosis of renal insufficiency indicated by serum creatinine ≥ 132 *μ*mol/L (≥1.5 mg/dL) in male patients and ≥123 *μ*mol/L (≥1.4 mg/dL) in female patients. Patients were also excluded in the event of pregnancy or lactation [11], severe infection, or significant neurological or psychological illness (depression, anxiety, and schizophrenia) that may influence the thyroid function and also those who used considerable quantity of hormones (sex hormones, glucocorticoid, or mineralocorticoid), which may directly or indirectly contribute towards the susceptibility to thyroid disease [12]. Finally, 201 patients with thyroid nodule disease (designated as the thyroid nodule group) and 308 patients without the nodular thyroid disease (referred as the control group) were included in the study.

This study was approved by the Ethics Committee of Tianjin Metabolic Diseases Hospital and conducted in accordance with the provisions of the Declaration of Helsinki.

### 2.2. Laboratory Assays and Ultrasound Measurements

All the patients underwent a physical examination of height, weight, and blood pressure. BMI was calculated as weight in kg/height in m^2^. The laboratory evaluation of glycated hemoglobin typeA1c (HbA1c, %), fasting plasma glucose (FPG, mmol/L), fasting insulin, triglycerides (TG, mmol/L), total cholesterol (TC, mmol/L), alanine aminotransferase (ALT, IU/L), aspartate aminotransferase (AST, IU/L), serum creatinine (Scr, umol/L), blood urea nitrogen (BUN, mmol/L), and uric acid (UA, umol/L) was measured following a minimum fasting period of 12 h. HOMA-IR was calculated according to the FPG and fasting serum insulin, recognized as simple and effective in evaluating the degree of insulin resistance index. HOMA-IR = FPG (mmol/L) × FINS (mIU/L)/22.5 [13]. The thyroid function evaluation index was evaluated by measuring the free FT4, free FT3, serum thyroid stimulating hormone (TSH), and thyroid peroxidase antibody (TPO) by the immunochemiluminescent assays, on an automated analyzer. All the participants underwent thyroid ultrasonography that utilized a 7.5 MHz linear probe (Logiq 3, GE Medical Systems, WI, USA). The diameter of the nodules was measured in three dimensions. The thyroid nodule volume was calculated using the formula for the volume of a prolate ellipse (0.523 × length × width × diameter) [14]. The largest diameter was recorded as the index of the thyroid nodule size [15]. Irrespective of the number of nodules, only the largest diameter and nodule volume were taken into consideration. All the ultrasonographic evaluations were performed by the same radiologist, blinded to the samples.

### 2.3. Statistical Analysis

The data were expressed as mean ± standard deviation (SD). The independent samples *t*-test was used to compare the differences in the clinical characteristics between the thyroid nodule and the control groups. The chi-square test was used for nonparametric variables. In the multivariate binary logistic regression analysis, the predictors of the increased volume and size of thyroid nodule were selected based on both clinical and statistical significance. Pearson's correlation analysis was used for evaluating the correlation between the thyroid nodule volume and size. The predictors of the thyroid nodule include age, gender, thyroid function evaluation index, and HOMA-IR. The independent samples *t*-test was used for comparing the differences in the predictors of increased thyroid nodule, volume, and size between males and females in the thyroid nodule group. The data were analyzed by SPSS software (Statistical Package for the Social Sciences, version 22.0, Chicago). *P* < 0.05 was considered statistically significant.

## 3. Results

The clinical characteristics of the subjects were described in Table 1. The present study included a total of 509 T2DM cases (201 patients in the thyroid nodule group and 308 patients in the control group). The two groups were well balanced at baseline regarding BMI, WC, blood pressure, and all the biochemical parameters including HbA1c, FPG, TG, TC, ALT, AST, Scr, BUN, and UA. In our study, the thyroid nodules were less prevalent in males than in females as compared to the control group (50.7 versus 72.7%, *P* < 0.01). Age, duration of T2DM, serum FT4, and HOMA-IR were significantly higher in the thyroid nodule group than in the control group (59.13 ± 10.23 versus 52.30 ± 11.23, *P* < 0.001; 9.34 ± 7.78 versus 7.34 ± 6.22, *P* < 0.001; 17.04 ± 2.09 versus 16.59 ± 2.11, *P* = 0.019; 8.16 ± 7.79 versus 6.3 ± 9.34, *P* = 0.017, resp.). On the other hand, the serum FT3 was lower in the thyroid nodule group than in the control group (4.46 ± 0.53 versus 4.57 ± 0.61, *P* = 0.048, resp.). The TSH and TPO levels were similar between the two groups (Table 1).

The binary logistic regression analysis showed that age, sex, FT4, and HOMA-IR remained as the risk factors for thyroid nodule in the T2DM patients (*β* = 0.57, *P* < 0.001; *β* = 0.743, *P* < 0.001; *β* = 0.05, *P* = 0.001; *β* = 0.022, *P* = 0.048, resp.) (Table 2). Conversely, TSH and FT3 did not affect the thyroid nodule (Table 2).

**Figure 1:**
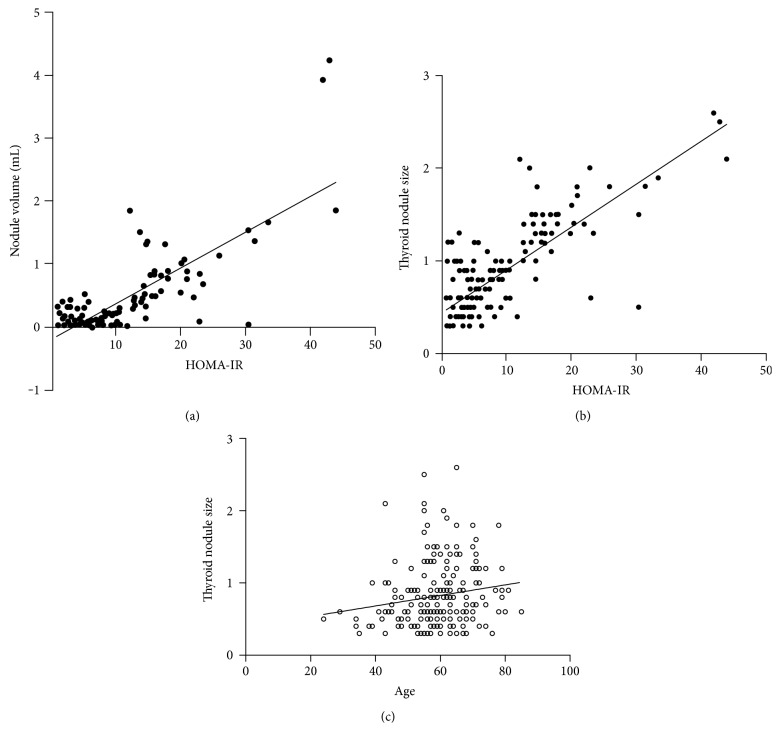
(a) The correlation between the thyroid nodule volume and HOMA-IR. A significant positive correlation between HOMA-IR and thyroid nodule volume in patients (*r* value: 0.808, *P* < 0.001). (b) The correlation between thyroid nodule size and HOMA-IR. A significant positive correlation between HOMA-IR and thyroid nodule size in patients (*r* value: 0.037, *P* < 0.001). (c) The correlation between thyroid nodule size and age. A significant positive correlation between age and thyroid nodule size in patients (*r* value: 0.167, *P* = 0.018).

Pearson's correlation analysis showed that the thyroid nodule volume and size were significantly positively correlated with HOMA-IR (*r* value: 0.808, *P* < 0.001; *r* value: 0.796, *P* < 0.001, resp.). Also, a positive correlation between age and thyroid nodule size was observed (*r* value: 0.167, *P* = 0.018). However, the thyroid nodule volume and size did not exhibit any correlation with the thyroid function evaluation index (Figure 1).

In the present study, the 201 patients with thyroid nodules encompassed 99 (49.3%) females and 102 (50.7%) males. The thyroid nodule volume and size were higher in females than in males in this group (0.40 ± 0.67 versus 0.18 ± 0.31, *P* = 0.003; 0.94 ± 0.50 versus 0.70 ± 0.35, *P* < 0.001). HOMA-IR and TSH were higher, whereas FT3 was lower in females than in males (9.42 ± 8.39 versus 7.02 ± 6.69, *P* = 0.026; 2.36 ± 1.06 versus 1.99 ± 0.91, *P* = 0.008; 4.34 ± 0.49 versus 4.58 ± 0.55, *P* = 0.001, resp.) (Table 3).

**Figure 2:**
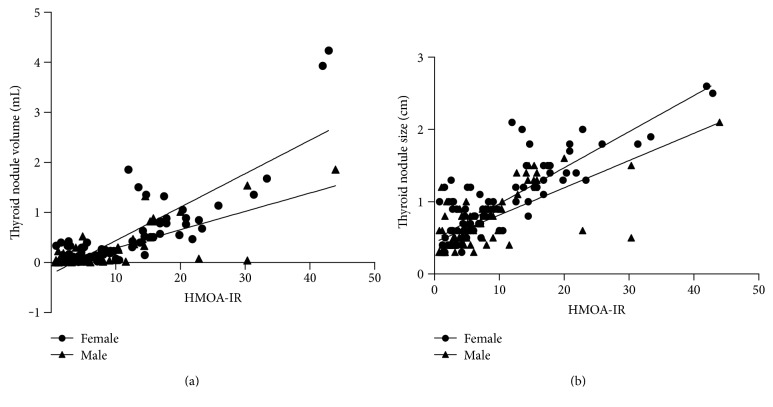
(a) The correlation between thyroid nodule volume and HOMA-IR in patients with type 2 diabetes mellitus with thyroid nodules. A positive significant correlation between HOMA-IR and thyroid nodule volume in female and male patients (*r* value: 0.842, *P* < 0.001 versus *r* value: 0.840, *P* < 0.001). (b) The correlation between thyroid size volume and HOMA-IR in the patients with type 2 diabetes mellitus with thyroid nodules. A positive significant correlation between HOMA-IR and thyroid nodule volume in female and male patients (*r* value: 0.780, *P* < 0.001 versus *r* value: 0.715, *P* < 0.001).

Pearson's correlation analysis showed that the thyroid nodule volume was significantly positively correlated with HOMA-IR in female and male patients (*r* value: 0.842, *P* < 0.001 versus *r* value: 0.708, *P* < 0.001) (Figure 2). Also, a significant positive correlation between HOMA-IR and thyroid nodule size was noted in both sexes (*r* value: 0.840, *P* < 0.001 versus *r* value: 0.715, *P* < 0.001) (Figure 2).

## 4. Discussion

Diabetes mellitus and thyroid nodule are the most common diseases in endocrinopathies of adult population. IR is one of the key factors in the pathogenesis of T2DM [16]. The prevalence of thyroid nodules has increased [17] along with the increasing prevalence of T2DM and IR globally in recent years. Thus, to establish a correlation between them, an in-depth investigation is essential. The thyroid nodule refers to the thyroid cells localized in the lesions caused by abnormal growth. All the factors that can bring about cell division may promote the thyroid nodule formation.

The glucose clamp technique is considered the “gold standard” to assess insulin sensitivity in humans, but is not adequate for studies involving hundreds or thousands of subjects in clinical investigations [18]. Bonora et al. demonstrated that HOMA is a valuable alternative to more sophisticated techniques in the evaluation of insulin sensitivity (or IR) in humans [13]. In the current study, we selected HOMA-IR as evaluation index of IR. Waist circumference, as one of the evaluation indices of IR, was also taken into consideration; however, statistically significant differences were not found between the thyroid nodule and control groups. Therefore, we chose HOMA-IR as the research target of IR for further analysis.

Anil et al. reported that the mean TSH level, thyroid volume, and the percentage of patients with thyroid nodules in the diabetes group were higher than the nondiabetes and prediabetes groups [7]. Junik et al. displayed that thyroid volumes were significantly higher in both subjects with T1DM and T2DM than the normal population [19]. T2DM patients were more likely to develop thyroid nodules as compared to type 1 diabetic patients and the nondiabetes group [19]. In our study, there were no differences between the thyroid nodule and control groups in FBG and HbA1c. We minimized the effect of blood sugar for the thyroid nodule and discussed the deeper influence factors of thyroid nodule in T2DM.

The occurrence of a thyroid nodule is determined by an interplay between genetic and environmental factors. A previous study showed that the thyroid nodules were common in women and usually increased with age and decreasing iodine intake [9]. In our study, the thyroid nodules were commonly found in females than in males, which was in agreement with the previous studies [9, 20]. The rate of prevalence of nodules was found to be 61.8% in the T2DM patients in Ankara (Turkey), which is a mild-to-moderate iodine-deficient area [7]. Tianjin is a coastal city, an iodine sufficient area, in the north of China with the nodule prevalence of 39.49%. Thus, we could eliminate the effect of iodine deficiency influence for the thyroid nodule in this cohort. Other factors such as tobacco smoking, pregnancy, use of estrogens, and alcohol consumption are also the risk factors underlying the formation of thyroid nodules [20]. In addition, the thyroid nodule has been reported to increase with age, which is considered as a risk factor for thyroid cancer [21]. Herein, we also found that age was higher in the thyroid nodule group than in the control group.

We found another positive indicator associated with nodule formation, HOMA-IR, which contributed to the volume and size of thyroid nodules. IR is a characteristic feature of most patients with simple obesity, polycystic ovarian syndrome, and impaired glucose tolerance, as well as hypertension, especially to T2DM [1]. Insulin-like growth factor-1 (IGF-1) is a major growth and differentiation factor for several cell types. IR is characterized by high blood glucose and insulin levels. The hormone insulin and insulin-like growth factor receptor (IGF-R) have been documented to play a key role in cancer biology, suggesting that insulin receptors are overexpressed in most tumors, including thyroid tumor, as an early step in several malignancies [22]. Some studies indicated that IGF-1 and IGF-1R are expressed by thyroid follicular cells and C-cells and play a role in cell regulation and proliferation in thyroid tissues [23]. An increase in insulin levels is advantageous in order to be able to interact the insulin with IGF-binding proteins (IGFBPs), thereby increasing the levels of free IGF-1 [24]. Pazaitou-Panayiotou et al. concluded that the ratios of IGF-1 to adiponectin and IGF-1 (adiponectin × IGF-binding protein 3 (IGF-BP3)) were positively associated with the tumor size in thyroid cancer patients [25]. Pitoia et al. found that IR is another factor that increases the risk of recurrence in patients with papillary thyroid cancer (PTC) [26]. In our study, the IR in the thyroid nodule group was higher than that in the control group. Using logistic regression analysis, we also substantiated that IR is the risk factor for the thyroid nodule in patients with T2DM. A similar study by Rezzonico et al. reported that patients with IR suffered a higher risk for the formation of a thyroid nodule and larger thyroid volumes as compared to the nondiabetic patients without IR [6]. The degree of IR and diabetic complications might be distinctly increased with the diabetes duration extension.

Some humoral or hormonal mediators, such as leptin, found to be increased in patients with T2DM [27], from adipose tissue, contribute towards the hypothalamus-pituitary-thyroid axis to elevate the TSH levels [28]. We found that the thyroid nodule group manifested similar BMI as compared to the control groups; thus, the leptin might not vary in the two groups. These negative findings might be attributable to another pathophysiological pathway function in T2DM. An alteration was often found in diabetic patients compatible with euthyroid sick syndrome (ESS) [29]. One of the most common is the reduction in FT3 in diabetic patients [30]. We found the thyroid nodule group with lower serum FT3 and higher FT4 concentrations than the control group. This supported the hypothesis of inhibited type 1 deiodinase and 5′-deiodinase activity, which is the primary pathway underlying the conversion of T4 conversion to T3 [31]. 5′-deiodinase inhibition may be related to sugar utilization obstacle inside the cell. Tumour necrosis factor (TNF) was significantly increased in type 2 diabetes compared to normal subjects that are associated with the ESS [32]. In van der Poll's study, TNF induced decreases in T3 and TSH levels, either directly or indirectly, in the pathogenesis of the ESS [33]. TNF can also impact 5′-deiodinase to reduce T4 convert to T3. Genetic abnormalities are shown to damage the expression and activity of type 2 deiodinase that has been associated with increased IR [34]. The activity of type 1 deiodinase may also be related to IR. On the other hand, the low FT3 and higher FT4 levels could be correlated with insulin levels, which could lead to decreased levels of thyroid hormone binding globulin [35]. Thus, the decreased and impaired synthesis of carrier proteins allied to the conversion of T4 to T3 would hypothetically result in a pattern of higher FT4 and lower FT3 [31]. The logistic regression analysis revealed that FT4 was a risk factor for the thyroid nodule in T2DM patients. Several studies have demonstrated that thyroid hormones such as FT3 and FT4 can influence the cell growth with respect to tumor and cancer cell proliferation [36]. However, in the current study, we observed that FT4 is associated with thyroid nodule.

The thyroid nodule volume and size, as well as HOMA-IR, were higher in females than males in T2DM patients with thyroid nodules. The elevated rate of incidence of thyroid nodule in females might be associated with the periodical changes in female endocrines [37]. According to Tu et al., the levels of T3 in the female thyroid nodule patients were higher than those in the males in 50 cases of diabetic patients [38]. Conversely, our results showed that the levels of T3 in the female thyroid nodule diabetic patients were lower those in males, along with higher levels of TSH. Accumulating evidence suggests that subclinical hypothyroidism (SCH) is more likely to occur in T2DM patients as compared to healthy individuals and may be associated with increased diabetic complications [39]. The American Association of Clinical Endocrinologists, Thyroid Disease Clinical Practice Guidelines recommends early treatment of subclinical thyroid dysfunction and requires regular screening for thyroid abnormalities in all diabetic patients [40]. Thus, the female T2DM with thyroid nodules potentially develops into subclinical or clinical hypothyroidism. Therefore, alleviating the IR might postpone this phenomenon in female patients with T2DM.

In a previous report by Anil et al., metformin therapy significantly decreased nodule size in subjects with IR [7]. Simultaneously, acarbose was superior to placebo in improving glycemic control and HOMA-IR [41]. The distribution of antidiabetic medications not only can treat diabetes but also reduce the volume and size of the thyroid nodule by improving IR. Further studies are required to treat the patients of thyroid nodule in T2DM with antidiabetic medications by improving IR.

## 5. Conclusion

Our study demonstrated that the insulin resistance was a risk factor for thyroid nodule in patients with T2DM. We concluded that the thyroid nodule volume and thyroid nodule size were increased with HOMA-IR in T2DM patients. The thyroid nodule volume and thyroid nodule size were larger in females than in males with T2DM, and they also increased with the level of IR. Alleviating the IR may decline their growth rate and minimize the volume and size.

## Figures and Tables

**Table 1:** Clinical, laboratory, and thyroid ultrasonography characteristics of study subjects (*n* = 509).

Variables	Thyroid nodule group (*n* = 201)	Control group (*n* = 308)	*t* or *χ*^2^ values	*P* values
Male (*n* (%))	102 (50.7)	224 (72.7)	25.521	<0.001
Age (years)	59.13 ± 10.23	52.3 ± 11.23	6.930	<0.001
BMI (kg/m^2^)	26.69 ± 3.64	26.60 ± 3.96	0.245	0.806
WC (cm)	95.81 ± 8.13	96.01 ± 8.41	−2.66	0.790
FBG (mmol/L)	8.63 ± 3.09	8.26 ± 1.96	1.661	0.097
HbA1C (%)	9.04 ± 2.04	9.33 ± 2.13	−1.576	0.116
ALT (IU/L)	21.80 ± 12.42	24.14 ± 16.34	−1.73	0.084
AST (IU/L)	18.59 ± 7.18	19.65 ± 9.59	−1.346	0.179
BUN (mmol/L)	5.45 ± 1.73	5.27 ± 1.61	1.251	0.212
Scr (*μ*mol/L)	63.86 ± 21.42	62.30 ± 15.77	0.947	0.344
UA (*μ*mol/L)	291.61 ± 81.94	294.26 ± 93.46	−0.328	0.743
TG (mmol/L)	2.17 ± 3.06	2.09 ± 2.07	0.378	0.706
TC (mmol/L)	4.82 ± 1.33	4.66 ± 1.19	1.424	0.155
FINS (mIU/L)	18.22 ± 9.39	13.95 ± 11.40	4.425	<0.001
HOMA-IR	8.2 ± 7.65	6.31 ± 9.25	0.159	0.016
TSH (*μ*IU/mL)	2.17 ± 1.00	2.08 ± 0.92	1.082	0.28
FT3 (pmol/L)	4.46 ± 0.53	4.57 ± 0.61	−1.981	0.048
FT4 (pmol/L)	17.04 ± 2.09	16.59 ± 2.11	2.363	0.019
TPOAb (IU/mL)	8.2 ± 7.65	6.31 ± 9.25	−0.8	0.424
Thyroid nodule volume (mL)	0.29 ± 0.53	—	—	—
Thyroid nodule size (cm)	0.82 ± 0.45	—	—	—

Values are expressed as mean ± SD or *n* (%). BMI: body mass index; WC: waist circumference; FBG: fasting blood glucose; HbA1c: glycated hemoglobin typeA1c; ALT: alanine aminotransferase; AST: aspartate aminotransferase; BUN: blood urea nitrogen; Cr: creatinine; UA: uric acid; TG: triglyceride; TC: total cholesterol; FINS: fasting insulin; HOMA-IR: homeostasis model assessment insulin resistance; TSH: thyroid stimulating hormone; FT4: free thyroxine; FT3: free triiodothyronine; TPO: thyroid peroxidase antibody.

**Table 2:** Logistic regression analysis for predictors of thyroid nodule.

Items	*β* values	SE	OR	95% CI	*P* values
Age	0.059	0.01	1.061	1.040–1.082	<0.001
Gender	0.730	0.210	2.076	1.376–3.132	0.001
TSH	0.018	0.106	1.018	0.828–1.253	0.863
FT3	−0.149	0.180	0.862	0.605–1.227	0.410
FT4	0.162	0.05	1.176	1.067–1.296	0.001
HOMA-IR	0.041	0.014	1.042	1.013–1.071	0.004

TSH: thyroid stimulating hormone; FT4: free thyroxine; FT3: free triiodothyronine; HOMA-IR: homeostasis model assessment-insulin resistance.

**Table 3:** The comparison of the characters between male and female T2DM patients with thyroid nodule (*n* = 201).

Variable	Male (*n* = 102, 50.7%)	Female (*n* = 99, 49.3%)	*t* values	*P* value
Age (years)	58.46 ± 11.03	59.82 ± 9.35	−0.94	0.348
TSH (*μ*IU/mL)	1.99 ± 0.91	2.36 ± 1.06	−2.663	0.008
FT3 (pmol/L)	4.58 ± 0.55	4.34 ± 0.49	3.306	0.001
FT4 (pmol/L)	17.05 ± 2.15	17.04 ± 2.05	0.034	0.973
TPO (IU/mL)	9.42 ± 4.74	9.35 ± 4.38	0.104	0.918
HOMA-IR	7.02 ± 6.69	9.42 ± 8.39	−2.245	0.026
Thyroid nodule volume (mL)	0.18 ± 0.31	0.40 ± 0.67	−2.978	0.003
Thyroid nodule size (cm)	0.70 ± 0.35	0.94 ± 0.50	−2.926	<0.001

HOMA-IR: homeostasis model assessment insulin resistance; TSH: thyroid stimulating hormone; FT4: free thyroxine; FT3: free triiodothyronine; HOMA-IR: homeostasis model assessment-insulin resistance.
